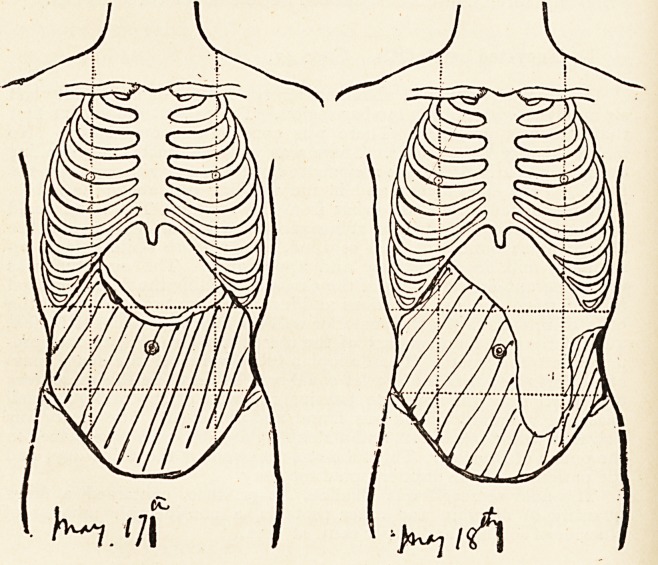# Fifty Consecutive Intra-Abdominal Operations

**Published:** 1898-09

**Authors:** James Swain

**Affiliations:** Professor of Surgery at University College, Bristol; Assistant-Surgeon to the Bristol Royal Infirmary


### FIFTY CONSECUTIVE INTRA-ABDOMINAL OPERATIONS.

BY

James Swain, M.S., M.D. Lond., F.R.C.S. Eng.,

*Professor of Surgery at University College, Bristol;*
*Assistant-Surgeon to the Bristol Royal Infirmary.*

By taking a retrospective view of a consecutive series of cases a better practical lesson is enforced than by the consideration of a larger number of mixed statistics grouped according to the particular fad of the statistician, and often devoid of any material benefit to the operating surgeon.

The term "intra-abdominal operation" has been preferred to "abdominal section," in order that such operations as herniotomy, nephro-lithotomy, &c. (which rarely require intra-abdominal manipulation of the kind here meant), may be excluded.

In the more difficult cases referred to the mortality is necessarily higher than if all abdominal sections had been included; but, having regard to the nature of the diseases and operations in the appended list, the percentage of deaths is by no means large.

This, however, is not a matter for congratulation, for the number of "late operations" is mainly responsible for most of the deaths; and so long as the surgeon is regarded as the *dernier ressort* in the treatment of acute intestinal obstruction, œsophageal stricture, appendicitis, malignant disease of the rectum and such like conditions, so long shall we have to be content with results which are far short of what can be accomplished by our present knowledge, and so long will an unnecessary waste of human life continue.

Every one knows that the mortality of operations for strangulated hernia increases rapidly with the number of hours that the gut has been strangulated, and the same thing is true in acute intestinal obstruction; and yet it is no uncommon thing to wait for the advent of fæcal vomiting before the obstruction is considered "dangerous."

The progress of malignant disease of the œsophagus is more insidious: and if the patient delays operation, as he often does, until life is maintained only by nutrient enemata, gastrostomy is of very doubtful expediency; for the mortality is then so high that the scalpel of the surgeon is little more than a means of inscribing the certificate of death on the wasted frame of the unfortunate patient.

It is not surgery, but the earlier need of it, that determines the death in many of these cases; and, highly desirable as it is to conscientiously avoid an unnecessary operation, it is equally highly desirable to urge an operation at the earliest possible moment where such operation is necessary.

In the following table the cases have been placed in the order of their occurrence, and no attempt has been made at classification. The term "death" is not synonymous with operative failure in the strict sense, but is used rather—and this is the most important—from the point of view of the ultimate result to the patient. Thus, in cases 5 and 47 the patients lived for about a fortnight, and each operation, which granted a measure of relief, was *quâ* operation quite successful, and in no way contributed to the "death" which subsequently ensued.

| No. | Sex and Age. | MEDICAL ATTENDANT. | DISEASE. | TREATMENT. | RESULT. |
|---|---|---|---|---|---|
| 1. | F. 19 | Mr. E. H. Openshaw. | Ovarian adeno-cystoma | Ovariotomy (right) .. | Cured |
| 2. | F. 32 | Mr. H. F. Devis ... | Hydrosalpinx ... ... | Removal of right ovary and tube | Cured |
| 3. | F. 61 | Mr. J. S. Griffiths ... | Cholelithiasis ... ... | Cholecystostomy ... | Relieved |
| 4. | F. 32 | Dr. E. F. Martin ... | Perforating gastric ulcer | Suture of ulcer ... | Cured |
| 5. | F. 43 | Dr. Aust Lawrence ... | Myo-fibroma of uterus; intestinal obstruction | Enterostomy ... ... | Death |
| 6. | F. 53 | Dr. G. F. Rossiter ... | Appendicitis ... ... | Drainage ... ... ... | Death |
| 7. | F. 38 | ... | Cystic ovary ... ... | Oophorectomy (right) | Cured |
| 8. | M. 12 | Dr. W. R. Awdry ... | Appendicitis ... ... | Drainage ... ... ... | Death |
| 9. | F. 58 | Mr. F. J. C. Parsons.. | Intestinal obstruction.. | Breaking down adhesions; release of hernia | Cured |

| No. | Sex and Age. | MEDICAL ATTENDANT. | DISEASE. | TREATMENT. | RESULT. |
|---|---|---|---|---|---|
| 10. | F. 56 | | Malignant stricture of œsophagus | Gastrostomy | Death |
| 11. | F. 38 | Mr. J. Ewens | Appendicitis | Appendicectomy | Cured |
| 12. | M. 32 | | Appendicular colic | Contents of appendix emptied into cæcum | Cured |
| 13. | M. 28 | Dr. W. Murray | Appendicitis | Drainage | Cured |
| 14. | F. 45 | Dr. A. Harvey | Carcinoma of pylorus | Exploratory incision | Unrelieved |
| 15. | F. 5 | Mr. J. Dacre | Hepatic abscess | Drainage | Cured |
| 16. | F. 47 | | Ovarian adeno-cystoma | Ovariotomy (left) | Cured |
| 17. | M. 22 | | Malignant stricture of rectum | Inguinal colostomy as a preliminary to Kraske's operation | Relieved |
| 18. | F. 27 | Mr. H. F. Devis | Pelvic abscess | Evacuation; immediate closure | Cured |
| 19. | F. 41 | Mr. J. F. Fry | Myo-fibroma of uterus. | Hysterectomy | Death |
| 20. | F. 42 | Mr. F. J. C. Parsons. | Intestinal obstruction. | Enterostomy | Death |
| 21. | F. 33 | Dr. H. Skelton | Chronic peritonitis | Lavage of peritoneum | Cured |
| 22. | F. 74 | Dr. E. Crossman | Malignant stricture of rectum | Inguinal colostomy | Death |
| 23. | M. 12 | Dr. J. C. Maclean | Appendicitis | Appendicectomy | Death |
| 24. | F. 44 | Dr. J. C. Maclean | Myo-fibroma of uterus | Hysterectomy | Cured |
| 25. | F. 23 | Mr. D. H. Forty | Appendicitis | Drainage | Cured |
| 26. | F. 17 | Mr. F. W. Friend | Appendicitis | Drainage | Cured |
| 27. | F. 24 | Mr. C. Bernard | Acute tubercular peritonitis | Exploratory incision | Death |
| 28. | F. 29 | Dr. J. M. Rattray | Retro-peritoneal tumour | Exploratory incision | Unrelieved |
| 29. | F. 30 | Mr. W. Danne | Retroversion of uterus. | Cœlio-hysteropexy | Cured |
| 30. | M. 59 | Mr. J. Dacre | Malignant stricture of œsophagus | Gastrostomy | Death |
| 31. | F. 45 | Mr. J. F. Fry | Myo-fibroma of uterus. | Hysterectomy | Cured |
| 32. | F. 54 | Dr. J. C. Maclean | Malignant disease of kidney | Exploratory incision | Unrelieved |
| 33. | F. 40 | Dr. E. G. Hall | Appendicitis | Appendicectomy | Cured |
| 34. | M. 11 | Mr. J. Dacre | Appendicitis | Drainage | Cured |
| 35. | F. 30 | Dr. F. H. Edgeworth. | Perforative peritonitis (? broad ligament abscess) | Drainage | Cured |
| 36. | F. 43 | Mr. J. H. R. Pigeon. | Perforating gastric ulcer | Irrigation of abdomen | Death |
| 37. | M. 50 | Mr. A. W. Clarke | Appendicitis | Drainage | Cured |
| 38. | M. 51 | Mr. E. J. Dore | Malignant tumour of colon | Colostomy; enterectomy | Death |
| 39. | F. 32 | | Retroflexion of uterus. | Cœlio-hysteropexy | Cured |
| 40. | F. 9 | | Appendicitis | Drainage | Death |
| 41. | F. 47 | | Ovarian adeno-cystoma: procidentia uteri | Ovariotomy; Cœlio-hysteropexy | Cured |
| 42. | M. 26 | | Appendicitis | Drainage | Death |
| 43. | F. 19 | | Encysted peritonitis | Flushing of cavity; immediate closure | Cured |
| 44. | F. 18 | Dr. W. Brown | Intussusception | Enterectomy | Death |
| 45. | F. 66 | Mr. J. Dacre | Ovarian adeno-cystoma (bilateral) | Ovariotomy (double) | Cured |
| 46. | M. 45 | Mr. H. Ormerod | Appendicitis | Drainage | Death |
| 47. | M. 71 | Dr. S. Irwin | Intestinal obstruction., | Enterostomy | Death |
| 48. | M. 57 | Dr. H. Skelton | Malignant stricture of rectum | Inguinal colostomy | Relieved |
| 49. | F. 47 | Mr. G. A. Brown | Hydatid disease of liver | Removal of endocyst; drainage | Cured |
| 50. | F. 20 | Dr. H. A. Benham | Appendicitis | Drainage | Cured |

In discussing these cases, those of a similar nature will be grouped together; but details will be given only in the more important ones, and comment made where desirable.

A. **Diseases of the ovaries and Fallopian tubes.** Cases 1, 2, 7, 16, 41, 45.

Of these cases 1, 16, 41, and 45 were adeno-cystomata.

CASE 1.—The vermiform appendix was adherent to the pedicle of the cyst, and required separation before the application of the ligature to the pedicle.

This is not a common source of danger in ovariotomy, but it exemplifies the necessity for thorough examination of the place chosen for the application of the ligature.

CASE 41.—This was a very large tumour, associated with complete procidentia uteri. The uterus was entirely outside the vagina, much hypertrophied, and about five inches in length. After ovariotomy the uterus was fixed to the abdominal wall.

It is very rare for the intra-abdominal pressure caused by ovarian adenoma to be sufficiently great to cause extrusion of the uterus. The case has been fully reported elsewhere,[1] and the question of hysteropexy for uterine displacement is considered below.

CASE 45.—There had been a gradual and painless enlargement of the abdomen for eighteen months, but associated with a good deal of pain on the right side of the abdomen for one week. The swelling presented the usual characters of ovarian adeno-cystoma. On the right side of the abdomen a peculiar friction rub could be felt and heard during the movement caused by respiration. Both ovaries were cystic, the left cyst containing about twelve pints of fluid. The pedicle of the left cyst was twisted about half a turn to the right, and a patch as large as the open hand was found in the cyst-wall of a dull white colour, devoid of vessels, and with a slightly roughened surface.

The patch referred to was the commencement of sloughing of the cyst-wall, and its roughened surface explained the friction rub which was felt and heard. Twisting of the pedicle is a very fatal complication of ovarian cystoma. The occurrence of pain in association with this disease should always be regarded seriously, and operation should not be delayed.

The most useful method of tying the pedicle in ovariotomy

[1] *Brit. M. J.*, 1897, ii. 399.

is by means of the "Staffordshire knot" with No. 4 plaited silk; but where the pedicle is very broad it must be secured by double transfixion and interlocking threads.

CASES 2 and 7 were allied in clinical symptoms. Both had severe pain, especially during the menstrual periods, and were reduced to a condition of chronic invalidism. Case 2 had suffered from many attacks of severe perimetritis. Case 2 was one of hydro-salpinx, and case 7 one of oöphoritic cyst. Each patient suffered from endometritis—which was probably the starting point of the disease—and a soft rounded swelling could be felt on the right side of the uterus in each case. Operation in cases of salpingo-oöphoritis requires careful consideration. Many cases get well, as shown by the fact that hydro-salpinx is never found in autopsies on old women. Expectant treatment is correct in the early stages; but where this has been duly tried, and the patient suffers from repeated attacks of inflammation and pain, with generally impaired health in consequence, operative measures are justifiable, and in most cases desirable.

B. **Myo-fibroma of uterus.** Cases 19, 24, 31.

All of these were of the "soft" variety, and were growing more or less rapidly.

CASE 19.—Patient had noticed a tumour in the abdomen eight or nine years; but it had increased in size very rapidly during the past two years, so that at the time of examination the abdomen was as distended as at full term of pregnancy, and the bulk of the tumour prevented the patient from attending to her household duties. Had suffered severely from menorrhagia. The incision for the removal of the tumour reached from just above the pubes to within two inches of the ensiform cartilage. The veins in the broad ligaments were as large as one's thumb, and the cæcum was adherent to the tumour. After removal, about three pints of blood escaped from the tumour, which then weighed 15½ lbs., and was 28¼ inches in the transverse and 30 inches in the vertical antero-posterior circumference. Great collapse followed the operation, and infusions of saline fluid were used, but the patient died of shock the morning after the day of operation.

Unfortunately, it is not possible to guard against death from shock due to the abstraction of a large amount of blood from the circulation in the operative treatment of such big fibroids as this, and the extra risk in such cases must be clearly stated before undertaking their removal.

Fibroid tumours occurring between thirty and forty years of age often grow rapidly, and the incidence of the menopause—which is usually delayed in these cases—does not necessarily inhibit their growth. Apart from mere bulk some form of operation is generally desirable in these tumours where there is rapidity of growth and the patient steadily loses more strength at each menstrual period than is regained in the interval. It is worth remembering, as Matthews Duncan has said,[1] that "a woman with an enormous fibroid will not live to be an aged woman."

In all the above cases the pedicle was treated by the extra-peritoneal method, and in only one of them should I have felt justified in adopting the intra-peritoneal method. The reasons for and against the two procedures cannot be here dwelt upon; but the "ideal" method is that by which the operator can get his patient well, rather than that which consists of a skilful surgical exercise at the patient's expense.

C. **Cœlio-hysteropexy for uterine displacements.** Cases 29, 39, 41.

Cases 29 and 39 were both the subjects of retroflexion, and the latter was also associated with some prolapse. Both had almost constant pain in the pelvis, worse at the menstrual periods, and were unable to wear pessaries without great discomfort. Occasional attacks of perimetritis and peri-oöphoritis had occurred, and in each case some thickening could be felt in the neighbourhood of a fixed, tender, and displaced ovary. After abdominal section the adhesions were broken down and the uterus sutured to the anterior abdominal wall. Both cases were entirely relieved of their pelvic pain.

Case 41 was a severe procidentia uteri associated with ovarian adeno-cystoma, and has already been referred to.

Cœlio-hysteropexy has little risk, and is suitable for such cases as those referred to. Adhesions can be dealt with satisfactorily by this method, and it, therefore, has a more extensive application than shortening the round ligaments (Alexander's

[1] *Clinical Lectures on Diseases of Women*, 3rd Ed., 1886, p. 330.

operation), which can only be employed in cases free from adhesions—a condition which is by no means easy of diagnosis, for I have found adhesions present at the time of operation where they were not suspected before. Cœlio-hysteropexy resolves itself into a suspension of the uterus, and if the fixation sutures be placed through the anterior surface just below the fundus—and not through the fundus itself (as is commonly done)—there is practically no risk of abortion in the event of pregnancy occurring, for the adhesions stretch as the uterus enlarges, and the fundus uteri itself is not dragged upon. The fixation sutures, generally three in number, should be left in for three weeks.

D. **Encysted peritonitis.** Case 43.

CASE 43.—Swelling of abdomen for two years—more rapidly last six months—with pain in lumbar region. The patient thought at first that she was pregnant. There was considerable emaciation. No family history of phthisis. There was a large fluctuating swelling occupying nearly the whole abdomen; but there was resonance in the flanks, in the epigastrium, and in the mid-line just above the pubes. This resonance varied somewhat from day to day. The hand could not be pressed back to the spine above the pubes. On opening the abdomen 222 ounces of fluid escaped, of a smooth white colour, resembling milk in consistency and appearance. The omentum was very adherent, and the walls of the cavity containing the fluid appeared to be formed of dense adhesions and inflammatory tissue amongst the coils of intestine. In the posterior wall the iliac vessels could be felt, and inferiorly the upper part of the uterus and broad ligaments projected into the cavity. The Fallopian tubes were lost in a dense mass of adhesions, so that the ovaries could not be felt. In the mesentery of a portion of the intestine forming the wall of the cavity a small caseating lymphatic gland was found and removed. The cavity was irrigated with boric lotion, and a Keith's glass drainage tube placed in the pouch of Douglas. This tube was removed on the third day, and the patient made an uninterrupted recovery.

The fluid removed was alkaline, sp. gr. 1020, contained a large quantity of albumin and other proteids, a considerable amount of chloride of sodium, and .6 per cent. of urea.

The appearance of the fluid at first suggested that I had to deal with one of those rare chyle cysts which occur in the abdomen; but the presence of the caseating lymphatic gland rather points to a tubercular origin. It is doubtful if the drainage tube need have been employed at all, for abscesses of this nature are generally free from such organisms as the staphylococcus pyogenes aureus and can be treated by immediate closure (*vide infra*). The case is reported mainly to show how a large fluctuating swelling in the abdomen may, before operation, closely resemble an ovarian cyst. The diagnosis was effected by two important signs present in this case. One was the resonance above the pubes, which scarcely ever exists in association with a large ovarian cystoma, and the other was the varying character of the relative positions of the dulness and resonance produced by the constantly changing amount of gaseous distension of the coils of intestine. This is exemplified by the following diagrams, in which the dull area on two successive days is roughly represented by the shaded portion.

It will, of course, be noticed that on each occasion the upper margin of the dull area was concave, which is the converse of that which obtains in the case of ovarian cystoma.

E. **Chronic peritonitis.** Case 21.

The pathology of many cases conveniently grouped together —for the sake of our ignorance—as chronic peritonitis is unknown. In this particular case it was thought desirable to treat it on similar lines to those adopted in chronic tubercular peritonitis, by which means the presence of a tumour could also be excluded.

Case 21.—Patient had noticed swelling of the abdomen for about sixteen months, for which she had been tapped about six times during the past six months. There was no tubercular history. Abdomen greatly distended; fluid thrill felt all over the abdomen, but no "tumour" could be felt. Liver dulness normal. A median incision was made, and 360 oz. of a yellow fluid (sp. gr. 1011, alkaline, highly albuminous) escaped. No cause for the peritonitis could be found. The abdominal cavity was flushed with boric lotion, and the wound closed without drainage. The abdominal cavity filled with fluid, and tapping had to be resorted to again; but after a third tapping there was some abdominal tenderness and temperature, and since then the patient has remained well.

The good results which so frequently follow operative procedures in cases of chronic tubercular peritonitis have been ascribed to various influences; but the above case, in which a cure did not result until definite inflammatory symptoms ensued, rather lends support to the theory that one of the chief causes of the improvement is the phagocytic action produced by operative traumatism.

F. **Acute tubercular peritonitis.** Case 27.

Case 27.—The patient was suddenly seized with abdominal pain and vomiting after a heavy meal of beef and pudding. The bowels acted after enemata only, and with difficulty. Five days after the commencement of the attack the vomit became stercoraceous. One sister died of phthisis, and father was suffering from same disease. The patient had an anxious expression. The abdomen was greatly distended tympanitic in front, dull in both flanks, but more on the right than the left side. General abdominal tenderness. On the fifth day of the disease an exploratory incision was made. On opening the peritoneum about twenty ounces of blood-stained fluid escaped. There was universal matting of the intestines, but the adhesions were easily separated in various directions, small quantities of fluid escaping from between the coils of gut. Both large and small intestines were of a bright red colour, being highly congested and swollen. On the parietal and visceral peritoneum were numerous discrete elevated milky-white, circular patches, varying in size from the head of a pin to that of a common tack. The vermiform appendix appeared normal. A drainage tube was inserted and the wound closed. After operation the vomiting ceased, and two good motions were passed spontaneously; but the patient gradually sank, and died three days later.

The chief interest in this case was in the diagnosis. It is unusual for tubercular peritonitis to set in so acutely. In another similarly acute case which I have seen there was, as in the above case, some difficulty in differentiating it from appendicitis. The case also bears out a clinical fact that I have noticed on several occasions; viz., that stercoraceous vomiting can occur without the presence of a mechanical obstruction.

G. **Acute peritonitis caused by the bursting of an ovarian or broad ligament abscess.** Case 35.

CASE 35.—The patient was suddenly seized with violent pains in the hypogastrium, and vomited. There was some collapse, but no abdominal distension. A third of a grain of morphia relieved the pain. Twelve hours afterwards a slight fulness of the abdomen was noticeable. There was general abdominal tenderness, most marked in the hypogastrium. Pulse 100. Temperature 101°. No diminution of liver dulness. An incision was made in the mid-line below the umbilicus, about fourteen hours from the commencement of the symptoms. Pus welled up behind the uterus from the left side of Douglas's pouch, but its exact source could not be determined. The appendix was normal. The pelvic cavity was gently washed with boric lotion, and a glass tube—removed in two days—was placed in Douglas's pouch. Recovery was rapid and uninterrupted.

There is frequently too great a tendency to temporize in such cases, the result being not uncommonly fatal. When the patient survives it is only after a severe and painful attack of pelvic peritonitis, which by its tendency to recur reduces the patient to a condition of chronic invalidism. Where symptoms of "perforation" are definite, as they were in this case, immediate operation holds out the best, and perhaps the only, chance for the patient. The actual cause of the perforation may not be evident, but this fact merely affords a stronger reason for surgical intervention.

H. **Pelvic abscess caused by sacro-iliac disease.** Case 18.

CASE 18.—For nearly two years there had been pain in the right hip, shooting down the thigh. Walking was difficult and painful, as the patient was scarcely able to bear any weight on the right lower limb. For two months gradually increasing swellings had been noticed on the right buttock and on the right side of the abdomen. The patient also suffered from a floating kidney, which easily slipped into place on pressure. Corresponding to the position of the posterior superior iliac spine was a swelling as large as the top of an egg. The swelling fluctuated, and had a distinct impulse on coughing. In the right lumbar and iliac regions of the abdomen was a large fluctuating swelling surrounded by considerable infiltration of the adjacent tissues, and extending from Poupart's ligament to the costal margin and from the outer border of the right rectus to the iliac crest. The two swellings communicated with each other, as fluctuation could be obtained from one to the other. On attempting to force the alæ of the ilia apart, considerable pain was felt in the right sacro-iliac joint. An incision over the posterior swelling disclosed the fact that the communication between the two abscess cavities was by means of the great sacro-sciatic foramen. This incision failed to allow of the necessary treatment of the anterior abscess, so after evacuating both cavities the wound was closed. After this the patient gained flesh and lost her pain; but the abdominal abscess gradually refilled. Two months after the former operation an incision was made over the abdominal swelling internal to the anterior superior iliac spine. The peritoneum was stripped up from the iliacus muscle, and on opening the cavity a large quantity of thin pus escaped. The abscess wall was thoroughly scraped with a Volkmann's spoon, and the cavity flushed with an izal lotion. The bones on either side of the sacro-iliac joint were bare for a distance of one inch. The wound was completely closed without drainage. A culture of the pus taken at the time of operation showed that the abscess was sterile. The patient made a complete recovery, and is now walking about in perfect health.

Sacro-iliac disease is generally credited with a very bad prognosis, and many cases die if the abscesses are treated by drainage tubes, for they then tend to become septic. This is obviated by the treatment above described, and the curettage and complete closure of such chronic abscesses cannot be too highly commended. The disease is thereby robbed of half its dangers, and the patient is spared the discomfort of wearing a tube. In psoas abscesses and those connected with disease of the hip-joint, I have adopted this method with the most gratifying results. It is interesting to note that this case was treated for a long time for sciatica before she came under my care. This is not an uncommon error in sacro-iliac disease, and is accounted for by the fact that the lumbo-sacral cord passes in front of the synchondrosis and causes the pain to be referred to the gluteal region.

I. **Operations on the liver.** Case 15, hepatic abscess; case 49, hydatid of the liver; case 3, cholelithiasis.

Case 15.[1]—The patient had suffered from a mild attack of enteric fever, followed by symptoms of a relapse. In about the eighth week of the disease from its commencement two rigors occurred, but for which no cause could be assigned until about a week later, when the cartilages of the seventh, eighth, and ninth ribs were found everted by a swelling in the left lobe of the liver which gradually increased in size. The swelling, which was tender on pressure, was regarded as a pyæmic abscess. During the latter part of the next fortnight a succession of rigors

[1] *Brit. M. J.*, 1898, ii. 149.

occurred, and the swelling in the liver enlarged rapidly, the lower edge being felt midway between the ensiform cartilage and the umbilicus. There was extreme emaciation; but as there were no evidences of pyæmic infarction in other organs, operation was urgently advised. A vertical incision was made over the prominent part of the swelling. An exploring needle was introduced, and some thin brownish pus withdrawn from an abscess cavity about half an inch from the surface of the liver. The parietal peritoneum was sewn to the surface of the liver, so as to shut off an area of about one inch in diameter. By tearing through the liver substance with sinus forceps, the pus was evacuated from a cavity the size of a cricket ball. A drainage tube was placed in the cavity. This was gradually shortened, and two months after operation the child had fully recovered her normal state of health. Agar cultures of the fluid taken at the time of operation were found to contain actively motile typhoid bacilli.

Hepatic abscess during typhoid fever is very rare and very fatal. I am not aware of any other case that has recovered, but the highly successful result in the above case should help to establish the desirability of surgical interference in this dangerous complication. Direct hepatotomy is generally to be preferred to operation *à deux temps*, both for abscess and hydatids. In the case just recorded the presence of some adhesions between the parietal peritoneum and that covering the liver greatly assisted the adoption of the operation in one stage.

Case 49.—This was an ordinary case of hydatid of the liver of five years' standing, the growth of the tumour being slow, painless, and unaccompanied by jaundice. The smooth fluctuating enlargement of the right lobe of the liver extended to the level of the umbilicus. Dulness over the swelling extended upwards as high as the fifth right rib. After exposure of the liver, over three pints of a clear fluid (sp. gr. 1010, alkaline, chlorides; no albumin) were drawn off. The cyst-wall was then incised, and the whole of the endocyst, which was loosely attached, was drawn out with forceps. There were no "daughter cysts." A drainage tube was placed in the cavity, and through this a free discharge of golden-yellow bile took place ten days later. The tube was finally left out at the end of five weeks, but the biliary fistula did not completely close for nearly six months.

The "hydatid fremitus" so carefully described in the textbooks was not present in this case: indeed, I have never felt it. Some cases have recently been reported[1] in which, after the removal of the endocyst, the opening in the ectocyst has been left open in the abdominal cavity and the parietal wound closed without any drainage whatever. If it becomes clearly established that this treatment is safe, the risk of sepsis will be lessened and convalescence much accelerated.

[1] *Austral. M. Gaz.*, 1898, xvii. 5.

Case 3.—For seven months the patient had been suffering from recurrent rigors, followed by jaundice. These attacks were accompanied by a temperature of about 102° for one or two days, and occurred about every week or ten days. In the intervals the jaundice would get less deep, but never entirely disappeared. There was great pain during the attacks, and the patient was profoundly cholæmic and steadily losing ground. The stools contained some bile, and the urine gave the reaction characteristic of bile pigments. Some resistance was felt in the right hypochondrium, but no definite enlargement of liver or gall-bladder could be made out. A vertical incision was made in a downward direction from the right tenth rib. Wide adhesions were found to exist between the gall-bladder, liver, and ascending colon. On passing the forefinger into the foramen of Winslow, several soft but enlarged lymphatic glands could be felt in the anterior part of the gastro-hepatic omentum. The ductus communis choledochus appeared somewhat enlarged, but on tracing it upwards from the duodenum no stone could be felt in it. The fundus of the gall-bladder was then incised; a slight amount of very thick brownish fluid escaped, and four facetted gallstones of medium size were extracted. The incision in the gall-bladder was sewn to the parietal wound, and the cavity drained by means of a rubber tube. The mucous membrane of the gall-bladder was much swollen and of a dark-red colour. Bacteriological examination of the fluid taken at the time of operation showed a very large number of bacilli and cocci. Many of the former were coliform organisms, and the latter were staphylococcus aureus. Since operation the patient has put on flesh, and had none of the attacks to which she was formerly subject. The final result, however, cannot be stated; for the biliary fistula had shown no tendency to close at the time of writing, and the necessity for cholecystenterostomy had arisen in consequence.

This case presented the now well-known symptoms of a stone in the common duct, but no such stone was found. The stone may have "floated" out of reach into one of the hepatic ducts, or—in common with a few other cases—the jaundice may have been due to the infective cholangitis which was obviously present, as shown by the condition of the mucous membrane of the gall-bladder, and the bacteriological examination of the contained fluid. Under such circumstances cholecystostomy is the best operation to adopt; as by this means the gall-bladder is drained, the cholæmia is relieved, and there is sometimes a chance of removing a stray stone through the fistula at a subsequent period. Where cholæmia exists in a marked degree, the mortality of operation is apt to be high. Not only is the cholæmia depressing, but there is a tendency to hemorrhage in such cases. For this reason chloride of calcium was administered for a few days before operation, as suggested by Mayo Robson. It is obviously important that operation should be undertaken before profound cholæmia supervenes. (The case passed out of my hands; but while this paper was going through the press I heard that cholecystenterostomy had been performed, and that at the time of operation a "floating" stone had popped up from the diverticulum of Vater, but disappeared upwards and could not be found again.)

K. **Gastrostomy.** Cases 10 and 30.

Both cases had only been able to swallow a small quantity of liquid for some weeks before operation. The œsophageal obstruction was eleven inches from the teeth in case 10, and nine and a half inches in case 30. The gastrostomy was performed by the Ssabanejew-Frank method in case 10, and by Greig Smith's method in case 30.

In nearly all cases of œsophageal carcinoma, the patient is greatly emaciated and much reduced in strength before he submits to operation. In such cases it is imperative that some method should be chosen which permits of the immediate feeding of the patient at the time of operation. Both the above operations give a good "water-tight" union of the stomach to the parietes, but of the two I am inclined to prefer the Ssabanejew-Frank method. It is rather simpler of performance, equally safe against leakage during feeding, and is more likely to prevent regurgitation of gastric contents on account of the obliquity of the opening into the stomach.

L. **Colostomy.** Cases 17, 22, 38, 48.

Cases 17, 22 and 48 were performed in the left inguinal region on the descending colon or sigmoid flexure. These three cases had malignant disease of the rectum. Case 17 was performed as a preliminary step to the removal of the growth by resection of the sacrum (Kraske's operation), which was done a fortnight later. Cases 22 and 48 did not admit of a radical cure of the disease. Case 38 was one of transverse colostomy for malignant disease of the transverse colon—the growth being excised four days after it had been brought to the surface.

In all the above cases the bowel was brought to the surface and kept up by means of a glass rod passing through the mesentery of the gut and resting at either end on the abdominal parietes (Reclus's method). This procedure is at once simple, speedy, and efficient; and as a routine operation (though not to be applied in all cases) for colostomy there is none better. Colostomy is, however, at the best an unsatisfactory operation. The patient is generally in the last stage of a mortal disease, and operation under the usual conditions is as likely to hasten, as it is to put off, the inevitably fatal issue. Where the disease cannot be removed completely, a palliative operation like colostomy should be performed before the patient is exhausted by the absorption of toxic products from intestinal accumulation. A common error is to suppose that the patient has not got obstruction because he complains of "diarrhœa," whereas this persistent diarrhœa should be regarded as the expression of an intestinal irritation set up by an overloaded colon which demands relief.

M. **Exploratory Operations.** Cases 14, 28, and 32.

Case 14 was one of carcinoma of the pylorus, in which the general condition of the patient and the extent of the tumour prevented the performance of pylorectomy.

Case 28 was an irremovable tumour (apparantly malignant) affecting the uterus, broad ligaments, and retro-peritoneal tissue.

Case 32 was a large malignant tumour of kidney, with secondary involvement of the parietal and visceral peritoneum.

None of these cases was made worse by exploratory incision, which is almost free from danger. In abdominal surgery treatment has outstripped diagnosis, and it is not infrequently necessary to get one's fingers in the abdominal cavity before an opinion can be fairly expressed as to the exact nature of the disease or the possibility of its removal.

N. **Perforating gastric ulcer.** Cases 4 and 36.

CASE 4.—Whilst bicycling the patient was suddenly seized with violent abdominal pain, beginning in the left hypochondrium. Some blood-stained and partially digested food was vomited, and the patient became collapsed. There was no previous history of any digestive trouble, except that in the spring of several preceding years she had had a little indigestion. Seven hours after the symptoms of perforation, I found the abdomen slightly retracted and the abdominal muscles rigidly contracted. Respiration was entirely thoracic, and of a superficial character. There was dulness in the splenic region downwards along the left flank, and coming forwards slightly towards the stomach. The usual liver dulness was replaced by resonance near the costal margin. An incision was made just below the ensiform cartilage. Free gas escaped on opening the peritoneum, and as operation proceeded about a pint of milky fluid with a few flakes of lymph escaped from the neighbourhood of the stomach and liver. A small perforation was found on the anterior surface of the stomach, rather nearer the cardiac than the pyloric end. Perforation closed with double layer of Lembert sutures. Irrigation with normal salt solution and drainage. One tube was placed between the liver and stomach, and another was made to drain the left kidney pouch through a counter opening in the left loin. The tubes were left out in about a fortnight, and the patient was able to take solid food in about three weeks.

Early operation and thoroughly efficient drainage are the essential conditions for success. The desirability of lavage of the peritoneum is a debatable one, but cannot be entered upon here. I have recently [1] raised this question in connection with another case. At present it will suffice to express the opinion that if lavage be adopted at all it should be carried out at low pressure, and over as limited an area as the conditions will allow. The stomach bears suturing well, but care should be taken to place the stitches sufficiently deeply to include a portion of the sub-mucous tissue. No. ½ or 1 plaited silk is the best suture material, and an ordinary milliner's needle, which is rounded and therefore does not cut the tissues, is the most useful for repairing all breaches of continuity in the stomach or intestines.

O. **Intestinal obstruction.** Cases 5, 9, 20, 44, and 47. (*Vide* also colostomy.)

These cases presented the usual difficulty in diagnosis. The cause of the obstruction was correctly diagnosed before operation in case 5 (large irremovable uterine tumour) and in case 9 (internal adhesions in the neighbourhood of an old hernia); but in case 44 (intussusception) and in case 47 (piece of bone above ileo-cæcal valve) the diagnosis was not made until after opera-

[1] *Brit. M. J.*, 1898, ii. 150.

tion; and in case 20 (Meckel's diverticulum) the cause remained undiscovered until the *post-mortem* examination. Several of these cases were not subjected to operation until very late. It may safely be said that in all cases of complete and acute obstruction the chances of recovery rapidly diminish for every hour that operation is delayed after the diagnosis is clearly established. Continuous pain and vomiting for forty-eight hours, accompanied, of necessity, by the deprivation of food, may so reduce the strength of the patient that the surgeon is unable to make that systematic examination of the contents of the abdomen which these cases frequently necessitate.

P. **Appendicitis.**—Cases 6, 8, 11, 12, 13, 23, 25, 26, 33, 34, 37, 40, 42, 46, and 50.

The fatal cases were all associated with a more or less general peritonitis existing before operation. Only one case (13) with this condition recovered.

Case 33.—The patient had been ill a fortnight with the usual symptoms. A firm indurated mass was felt in the right iliac fossa, above the outer third of Poupart's ligament. No fluctuation could be felt. Pulse 108. Temperature 101°. An incision was made over the indurated area just above Poupart's ligament. On opening the peritoneum an inflamed mass of agglutinated intestines was found. Boring amongst the intestines with the forefinger, I opened an abscess cavity, the walls of which were formed by the adherent coils of gut, and about two ounces of fœtid pus escaped. Projecting into this cavity was a rather short, thickened, and inflamed appendix. A collar of peritoneum was stripped back near the base of the appendix, the mucous and muscular coats ligatured and the terminal portion of the appendix cut away. The collar of the peritoneum was then closed over the invaginated stump. Iodoform gauze was placed in the abdomen to shut off the inflamed coils of intestine from the rest of the abdominal cavity, and a rubber tube was inserted, The temperature at once came down to normal. The tube was finally removed in about ten days, and the patient was thoroughly convalescent in three weeks.

Case 34.—Patient had been ill one week. A well-defined swelling could be felt in the right iliac fossa, above the outer half of Poupart's ligament and the anterior superior iliac spine. Deep fluctuation could be felt. Temperature 102°. On cutting down over the swelling about eight ounces of very foul pus escaped from a very large abscess cavity which passed down into the pelvis. At the upper part of the cavity, and fixed to its posterior wall, a thickened appendix could be felt; but being bound down and forming part of the abscess wall, it was not removed. Rubber tube inserted. The patient was collapsed for some time after operation, but in two days the temperature was normal, and he soon made a rapid recovery.

These cases are reported to show two classes of abscess which occur in appendicitis, and which differ considerably in their management. In the one (case 33) we find the appendix more or less free in a cavity formed by agglutinated intestines—and in such cases it is generally best to remove it, for the isolation of the appendix is a necessary part of the means taken for evacuating the abscess; in the other (case 34) the appendix itself forms part of the abscess wall, and in such cases an attempt to remove it would probably result in contamination of the general peritoneal cavity.[1] Many cases of the latter variety tend to approach the parietes to such an extent that the abscess can be opened without entering the general peritoneal cavity at all. For this reason it is always a good rule to keep the incision as far out as possible, and in searching for the abscess the tip of the finger is the safest instrument with which to bore amongst the inflamed and adherent tissues. I have several times operated upon cases with extensive suppuration in which the temperature has been normal, or only slightly raised above it. In these cases the tumid abdomen, the frequent pulse, and the drawn anxious expression tell their own tale of extensive abdominal mischief. This is not the place to consider the many vexed questions which surround the subject of appendicitis; but perhaps one may point out the desirability of constant watchfulness during the progress of a disease which may change its type at any moment, and place the patient in such danger that only immediate surgical interference holds out any prospect of life.

Want of space has prevented me from referring to many interesting clinical facts. The object of the paper is to bring forward those practical methods which I have personally found to be most useful in the treatment of cases which fall to the lot of an operating surgeon.

[1] See *Bristol M.-Chir. J.*, 1894, xii. 9; 1896, xiv. 336, where I have also dealt with this question.

## Figures and Tables

**Figure f1:**